# White stork movements reveal the ecological connectivity between landfills and different habitats

**DOI:** 10.1186/s40462-023-00380-7

**Published:** 2023-03-28

**Authors:** Cosme López-Calderón, Víctor Martín-Vélez, Julio Blas, Ursula Höfle, Marta I. Sánchez, Andrea Flack, Wolfgang Fiedler, Martin Wikelski, Andy J. Green

**Affiliations:** 1 Department of Wetland Ecology, Estación Biológica de Doñana CSIC, Seville, Spain; 2 Department of Conservation Biology, Estación Biológica de Doñana CSIC, Seville, Spain; 3 SaBio Health and Biotechnology Research Group, Institute for Game and Wildlife Research (IREC), CSIC-UCLM-JCCM, Ciudad Real, Spain; 4 Collective Migration Group, Max Planck Institute of Animal Behavior, 78315 Radolfzell, Germany; 5 Department of Migration and Immuno-Ecology, Max Planck Institute of Animal Behaviour, Radolfzell, Germany; 6 Centre for the Advanced Study of Collective Behaviour, University of Konstanz, 78468 Constance, Germany; 7 Department of Biology, University of Konstanz, Constance, Germany

**Keywords:** Connectivity, GPS tracking, Network analysis, *Ciconia ciconia*, Landfills, Wetlands

## Abstract

**Background:**

Connections between habitats are key to a full understanding of anthropic impacts on ecosystems. Freshwater habitats are especially biodiverse, yet depend on exchange with terrestrial habitats. White storks (*Ciconia ciconia*) are widespread opportunists that often forage in landfills and then visit wetlands, among other habitats. It is well known that white storks ingest contaminants at landfills (such as plastics and antibiotic resistant bacteria), which can be then deposited in other habitats through their faeces and regurgitated pellets.

**Methods:**

We characterized the role of white storks in habitat connectivity by analyzing GPS data from populations breeding in Germany and wintering from Spain to Morocco. We overlaid GPS tracks on a land-use surface to construct a spatially-explicit network in which nodes were sites, and links were direct flights. We then calculated centrality metrics, identified spatial modules, and quantified overall connections between habitat types. For regional networks in southern Spain and northern Morocco, we built Exponential Random Graph Models (ERGMs) to explain network topologies as a response to node habitat.

**Results:**

For Spain and Morocco combined, we built a directed spatial network with 114 nodes and 370 valued links. Landfills were the habitat type most connected to others, as measured by direct flights. The relevance of landfills was confirmed in both ERGMs, with significant positive effects of this habitat as a source of flights. In the ERGM for southern Spain, we found significant positive effects of rice fields and salines (solar saltworks) as sinks for flights. By contrast, in the ERGM for northern Morocco, we found a significant positive effect of marshes as a sink for flights.

**Conclusions:**

These results illustrate how white storks connect landfills with terrestrial and aquatic habitats, some of which are managed for food production. We identified specific interconnected habitat patches across Spain and Morocco that could be used for further studies on biovectoring of pollutants, pathogens and other propagules.

**Supplementary Information:**

The online version contains supplementary material available at 10.1186/s40462-023-00380-7.

## Background

Disentangling connections between habitats is crucial for understanding and predicting the impact of anthropic activities in ecosystems [38]. For instance, connectivity between habitats provided by animal movements can translate into eutrophication [37], heavy metal contamination [39], plastic pollution [60] and the dispersal of alien species [25] and pathogens [1].

In this context, the link between anthropic terrestrial habitats and wetlands is especially interesting for several reasons. First, the particular value of wetlands in terms of ecosystem services has long been recognized [30]. Second, wetlands are among the top priority habitats from a conservation point of view, since these habitats are declining worldwide as a consequence of anthropic pressure and climate change, especially in the Mediterranean region [2, 64]. Third, wetlands can hold particularly large numbers of birds concentrated within relatively small areas [23, 52]. Fourth, migratory waterbirds move frequently among different sites within a particular stage of their life cycle, and move considerably more than other avian groups [26, 38].

Many waterbirds, such as storks (*Ciconiiformes* [65]), are generalist foragers. The white stork (*Ciconia cionia*) forages intensively in landfills as they reduce energy expenditure by means of shorter foraging trips, less foraging time or increased foraging efficiency [19, 62]. This reliable food source has encouraged white storks to change stopover areas, to shorten migratory distances or even to suppress migration, leading to higher survival during migration [19]. As a consequence, the European population of white storks has increased dramatically since the 1980s [7, 24, 62]. This may lead to human-wildlife conflicts (e.g. [12] but also to ecological dis-services. When feeding on landfills, white storks ingest a wide range of solid waste such as plastics, metals, textiles or glass [7, 49], antibiotic resistant bacteria [28, 50] and resistance genes [31]. After having fed on landfills, white storks frequently use wetlands for resting among other habitats [7]. White storks also visit wetlands for additional foraging on prey such as fish [19] or the invasive Red Swamp Crayfish *Procambarus clarkii* [41, 42]. Consequently, white storks are acquiring pollutants in landfills, transporting them in their guts, and potentially depositing them within other habitats through their faeces, regurgitated pellets, and carcases [28, 49].

In addition to the foraging behaviour of white storks, owing to its migratory behaviour large numbers of individuals from different populations can concentrate at specific sites during certain periods of the year. Thus, the high abundance of this species when staging and wintering exacerbates its functional role in the ecosystem during these periods [6]. For example, white stork populations breeding from Portugal to Western Germany [4, 7, 19, 62] congregate in southern Spain and northern Morocco during migration or in winter.

Network applications have not been widely applied to spatial ecology compared to other fields such as social sciences or animal behaviour [20, 33, 59]. Nevertheless, network analysis is a powerful tool for quantifying connectivity between habitat patches [5, 6, 66]. Under a spatial network approach, specific sites used by animals to fulfil their biological requirements can be designated as nodes, whereas links emerge when individuals move between nodes [20, 38, 66, 67]. Traditionally, animal movement was estimated in absence of real quantification [20]. Nowadays, Global Positions System (GPS) datasets provide an exceptional framework to study real links between habitat patches by accurately quantifying individual movements [5, 46, 66]. The collection of GPS datasets in repositories such as Movebank [32] opens a new avenue of research for quantifying habitat connectivity.

The first objective of this study was to decipher the spatial network of sites used by white storks across all the Iberian Peninsula and Morocco. Secondly, we calculated centrality metrics and identified spatial modules within this network. Thirdly, we quantified the overall connectivity between habitat types. Our fourth objective was to explain the topology of the spatial network as a response to node habitat, focusing on the interface between landfills and aquatic habitats. For this, we used Exponential Random Graph Models (ERGMs) to quantify the effects of landfills acting as sources of movements and of wetlands acting as sinks.

## Methods

### White stork tracking data

We analysed movements of white storks wintering in the Iberian Peninsula and Morocco, for which we downloaded GPS data from Movebank [32]. Specifically, we used lifetracks from white storks tagged as chicks in southwestern Germany [14]. In all cases, we first filtered individuals that spent the winter within latitudes 30.0° N–42.5° N and longitudes 10.0° W–3.5° E, excluding those that wintered in Sub-Saharan Africa as they would bias the dataset to stopover areas [4, 38]. Second, we filtered all GPS positions during the non-breeding period, as defined from September to March [7]. Many individuals were present in the dataset for several years, so we consider a “bird-year” to be all consecutive positions of a given individual during a given non-breeding event. For example, the bird-year “3029–2017” represents all movements recorded by GPS tag “3029” from September 2017 to March 2018. We then calculated great circle distance, time difference and trajectory speed (km/h) between consecutive GPS fixes. In order to standardize sampling effort, we filtered positions separated by more than four minutes and less than 61 min. We also filtered the dataset by deleting position fixes with speeds higher than 100 km/h, because we assume that it is unrealistic that a given stork can fly so fast [11]. After applying these filters, we re-calculated distance, time difference and speed between consecutive fixes. Then, 90% of all fixes were separated by five minutes and 99% by up to 21 min. Position accuracy in our dataset was 10 m on average, and always lower than 100 m. Finally, we filtered our dataset for quality by removing specific bird-years with very few fixes (i.e. < 1000), because we assumed that such cases were driven by technical issues or early mortality. After applying these five filters, our dataset contained a total of 107 individuals and 204 bird-years (from 2013 to 2021).

### Site selection

We identified hotspots of GPS activity by overlaying our study tracks onto a land use surface [38, 66]. We used polygons from Corine Land Cover 2018 as the basis of our method [16]. For the purposes of this study, we often modified the shape of polygons and we also reclassified habitat categories (Table 1). In summary, we categorized 14 different habitats across our study area: three urban surfaces (landfills, urban areas, golf course), three agricultural terrestrial habitats (agro-forestry areas, non-irrigated arable land, permanently irrigated land), five artificial wetland habitats (rice fields, fish aquaculture, irrigation ponds, dams, salines) and three natural wetland habitats (marshes, lakes and ponds, water courses). To avoid overcomplicating the network, we joined close polygons of similar land use to create a single node if they were separated by up to 10 km. A similar merging operation was used by Merken et al. [43], and spatial autocorrelation for white storks breeding in southern Spain was estimated to be 9–12 km [12]. In the case of wetland polygons, we included a buffer around the shoreline because this is a favoured habitat for white storks. Many habitat patches across Spain (i.e. Andalusia, Extremadura and Castile-La Mancha) were ground truthed locally, and we assume that our habitat classification is also appropriate for Morocco where we could not ground truth due to logistic constraints.

**Table 1:** Reclassification of Corine Land Cover (CLC) categories to be used in this study

CLC code	CLC label	Label in this study
111	Continuous urban fabric	Urban areas
112	Discontinuous urban fabric	Urban areas
121	Industrial or commercial units	Urban areas
131	Mineral extraction sites*	Landfills
132	Dump sites	Landfills
142	Sport and leisure facilities	Golf course
211	Non-irrigated arable land	Non-irrigated arable land
212	Permanently irrigated land	Permanently irrigated land
213	Rice fields	Rice fields
244	Agro-forestry areas	Agro-forestry areas
411	Inland marshes	Marshes
421	Salt marshes	Marshes
422	Salines	Salines
422	Salines	Fish aquaculture
511	Water courses	Water courses
512	Water bodies	Lakes and ponds
512	Water bodies	Dams
512	Water bodies	Irrigation ponds

*Dump sites (i.e. landfills) were often wrongly classified by CLC as “Mineral extraction sites”. They may look similar from satellite images but we can recognize landfills by the concentration of GPS fixes [7, 24, 47]

In Morocco, Corine Land Cover is not available nor, to the best of our knowledge, is any other land cover layer with the required level of detail. Thus, we polygonized those sites of interest selected as potential nodes of our network by hand. We used the same habitat classification as given above and we examined up-to-date satellite images (https://www.sentinel-hub.com/explore/sentinelplayground) to identify temporary wetlands. We further improved our land cover layer for Morocco with published maps [68].

**Fig. 1:**
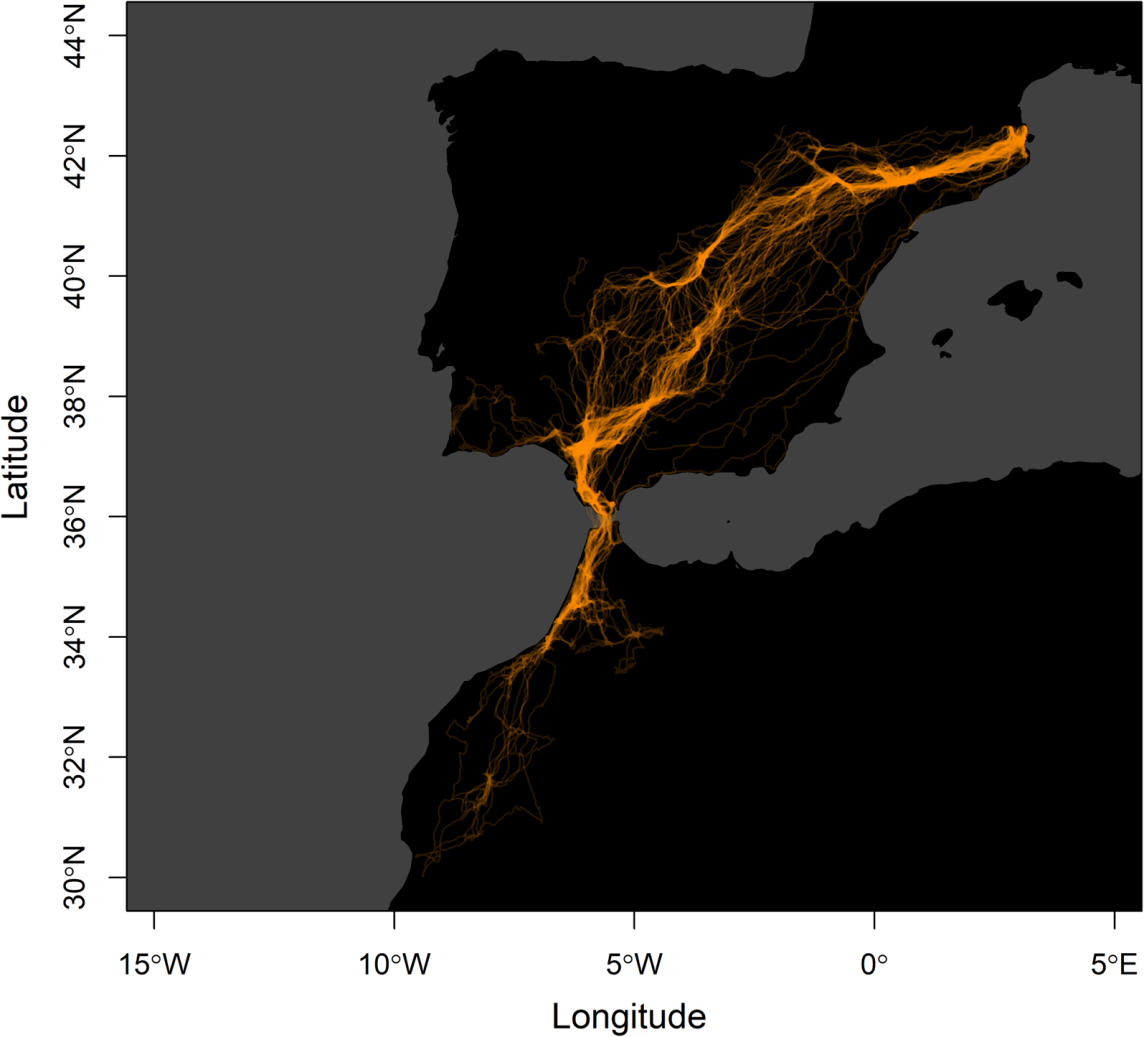
White stork tracks analysed in this study (103 individuals, 199 bird-years and 4,651,324 GPS fixes). Data are shown after applying five filters to the original GPS dataset downloaded from Movebank (see “Methods” section for details). White storks were tagged as nestlings in southwestern Germany and they all spent the winter either in the Iberian Peninsula or Morocco

All these steps were developed with software ArcMap 10.5, and we finally created a shape file with 181 potential nodes. We acknowledge that our method to select nodes is subject to error, and that a few specialist individuals could explain the formation of certain nodes [13]. Therefore, we filtered potential nodes based on the number of non-breeding white storks present. In order to do this, we calculated the number of bird-years that used each potential node and then we took the median value of bird-years per node as a cut-off criterion (for nodes with latitude 38° N or below including wintering areas in southern Iberian Peninsula and northern Morocco). In other words, we only retained as final nodes of our network these with six or more individuals in a given non-breeding event. After this final filter, we selected a total number of 114 nodes to build our spatial network. Such nodes were connected by 199 non-breeding events of 103 individuals. The final GPS dataset included 4,651,324 position fixes (Fig. 1), 83% of which were inside any node. Habitat class “Golf course” was removed in the final selection of 114 nodes.

### Observed network

Once we identified the nodes of our observed network, we created the links by filtering “direct flights” in the GPS dataset (i.e. non-stop flights). For this purpose, we adapted the R code developed by Martín-Vélez et al. [38]. We defined a direct flight from one node to another as the subset of consecutive positions during flight (fixes with speed > 10 km/h), beginning in the first node with speed < 10 km/h and ending in a different node with speed < 10 km/h. To filter a non-stop flight, we used 10 km/h as the cut-off criterion above which we assume that a given white stork is flying [11, 38]. Direct flights with any time difference between fixes higher than one hour were removed, to exclude trajectories with long data gaps. Thus, we defined a directed network weighted by the total number of direct flights in each link. After this process, we identified 40,447 direct flights distributed across 370 unique links (so called “edges”).

To continue with the second objective of our study, we calculated node betweenness as an index to identify key bottlenecks in the spatial network [5, 66]. Node betweenness quantifies the number of times a node acts as a bridge along the shortest path between two other nodes, relative to the total number of shortest paths between such nodes [17]. We weighted betweenness by the geographical distance among node centroids (in meters). Moreover we calculated strength, which is the alternative version of degree for weighted networks [33], and can be interpreted as an index to identify key nodes in the landscape acting as sources or sinks of movements [66]. In directed networks, the strength can be obtained for “in edges” *vs* “out edges”, being respectively the sum of all link values coming to a given node, or all link values leaving from the given node [17]. In addition, we determined modules within our spatial network, as defined by clusters of nodes highly connected between themselves and barely connected to others (i.e. “functional units” sensu [26, 38]). For this purpose, we used the method described by Rosvall and Bergstrom [54], which generates a particular number of random walks through the network (e.g. 1000) and then obtains the community structure that minimizes the expected description length of the random walk.

To fulfil the third objective of our study, we quantified the general connectivity between habitat types by summing direct flights for those links connecting the same habitats. In this way, we created an undirected network with 13 nodes (i.e. habitat types) and 55 links. We also built this habitat network separately for southern Spain (13 nodes and 32 links) and northern Morocco (9 nodes and 31 links). We made these additional networks to quantify the overall connectivity among different habitats, and no further analyses were conducted.

We used R packages *sp* [48] and *rgeos* [10] to process the spatial data. We built the network and calculated network metrics with R package *igraph* [17]. In order to facilitate detailed visualization of GPS tracks together with our spatial network, we developed an interactive map available at Additional file 1: Appendix S1. We used R package *leaflet* [15] for this task.

### Network modelling

The statistical analysis of networks should not be done under conventional approaches such as linear models because these methods assume independence of residuals, which is violated within a given network [59]. Link dependencies are common on landscape networks, such as movements across stepping-stones and along sites acting as sources or sinks [20]. Exponential Random Graph Models (ERGMs) are designed in direct analogy to the classical Generalized Linear Models (GLMs), but they enable hypothesis testing about the processes driving local network structure and link formation [29, 59]. More specifically, ERGMs for weighted networks allow testing the effects of different explanatory variables (so called “network statistics”) on a particular link value [35]. Modelling weighted networks with ERGMs requires establishment of a reference distribution, which should be given by the nature of the weight variable (in our case, Poisson). Using Poisson-reference ERGM creates the familiar log-linear effect, thus the final coefficient estimates indicate log-linear increase in our weight variable (direct flights). In this study, we used *R* packages *ergm* [29] and its extension for weighted networks *ergm.count* [35].

The topology of our full spatial network is affected by migratory behaviour of white storks tagged in Germany, which reached Spain after following the Gulf of Lyon during their southbound migration [19]. The nodes visited from Catalonia to Castile-La Mancha are frequently used as stopover sites on the route towards southern Spain and northern Morocco, where nodes are more densely connected [7, 19]. Therefore, we fitted two ERGMs: one for southern Spain (i.e. Andalusia, with 34 nodes) and another for northern Morocco (from Tanger to Rabat, with 31 nodes).

We aimed to estimate the effect of landfills and each wetland habitat on the number of direct flights at a particular link. Thus, we included in our models certain levels of the node factor “habitat”, adding a specific network statistic to the ERGM for each of these levels [45]. Because our network is directed, we included different network statistics to test the effect of certain habitats acting as sources (so called “out edges”) or sinks (“in edges”) of direct flights. The node factor included for out edges was habitat level “landfills” (i.e. the potential source of pollution), whereas node factors included for in edges were the eight classes of wetlands (i.e. potential sinks of pollution). We did not include fish aquaculture, irrigation ponds, dams or salines in the ERGM fitted for Morocco, since these habitats were not present in that network. As with classical GLMs, the effect estimated for each factor level represents the difference from the intercept (which includes all default levels).

In addition, we controlled the effects of covariates within our models. The sum of all link values, which is analogous to having an intercept within a GLM, controls that specific link values depend on neighbouring links [35, 59]. White storks, as large soaring birds, tend to move short distances between suitable habitat patches [19, 24] and consequently neighbouring nodes are expected to be more connected than distant ones [20]. To account for this spatial effect in our models, we included as a covariate the matrix of distances between node centroids. Finally, because white storks usually make repeated visits to the same foraging and resting sites [13], the number of direct flights from one node to another is expected to be similar for the reverse link. Therefore, we included mutuality as a covariate, expressed as the negative absolute difference [35].

## Results

### Observed network

**Fig. 2:**
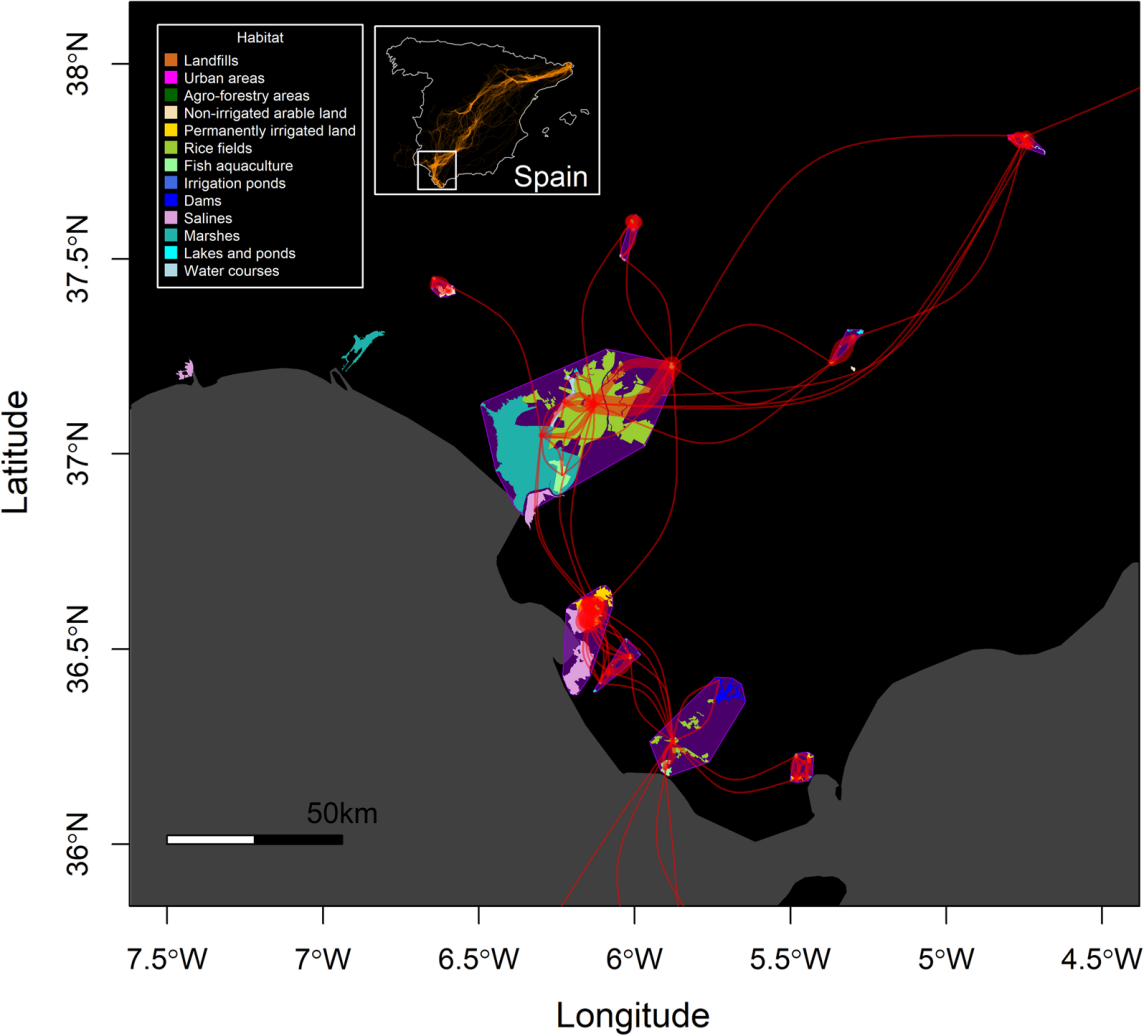
Spatial network of non-breeding white storks in southern Spain. Nodes are depicted as polygons, and colours indicate habitat types (see legend). All potential nodes used by less than six bird-years were removed from the network, so these sites are not connected to the rest (see “Methods” section for details). Links are represented as curved red lines, the wider the line the higher the number of direct flights. Modules are represented by convex hull polygons (coloured in violet) enclosing several nodes

**Fig. 3:**
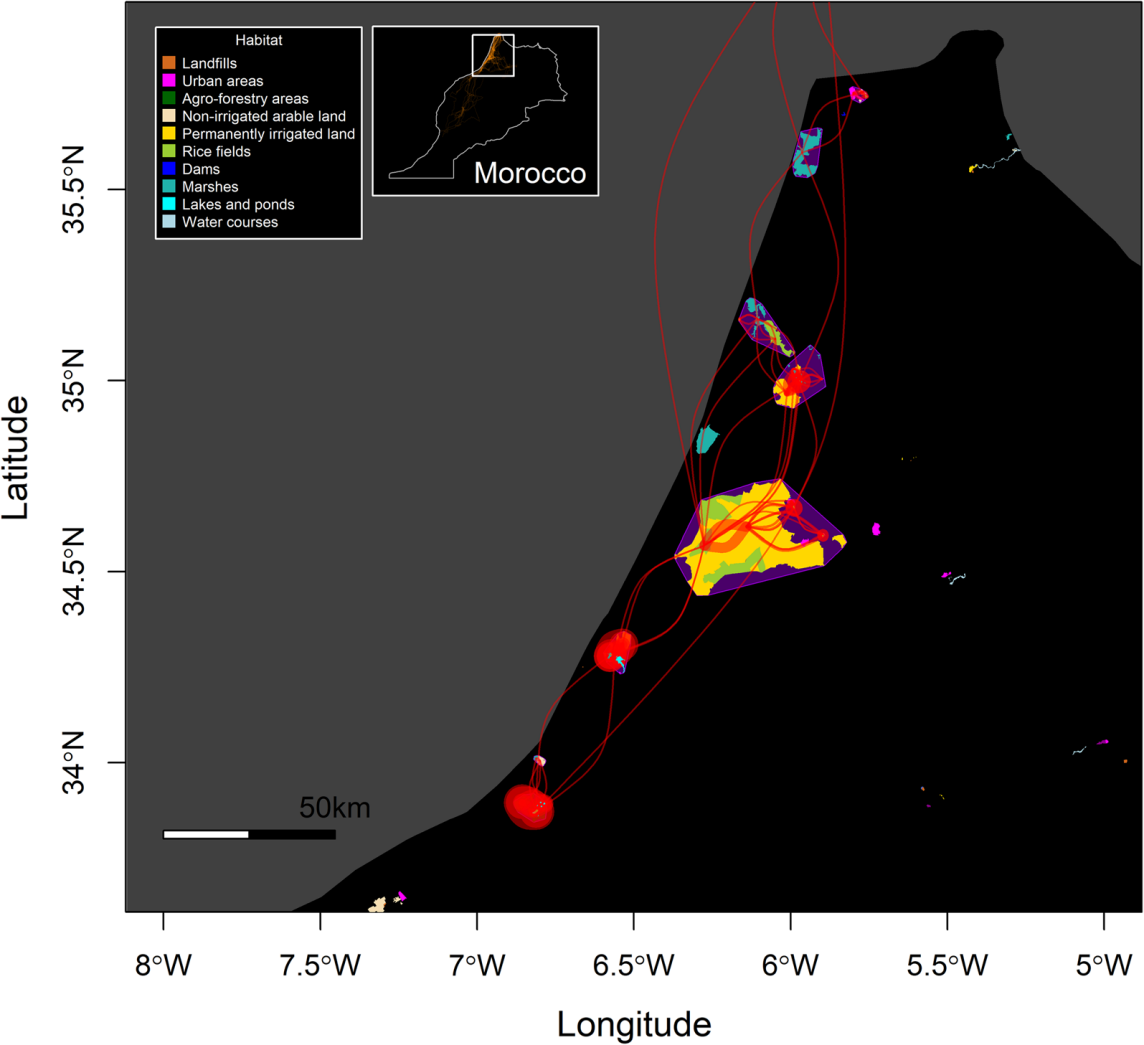
Spatial network of non-breeding white storks in northern Morocco. Nodes are depicted as polygons, and colours indicate habitat types (see legend). All potential nodes used by less than six bird-years were removed from the network so these sites are not connected to the rest (see “Methods” section for details). Links are represented as curved red lines, the wider the line the higher the number of direct flights. Modules are represented by convex hull polygons (coloured in violet) enclosing several nodes

By combining stork movement data with our site definitions across Spain and Morocco, we built a directed spatial network with 114 nodes and 370 links. Link values ranged from one to 2415 direct flights (mean ± SD = 109.31 ± 270.75). The full spatial network can be visualized at a site-by-site level in the interactive map (Additional file 1: Appendix S1). Figures 2, 3 show subsets of the observed network for southern Spain and northern Morocco, respectively.

The centrality measures we calculated for all nodes are given in Table S1 (Additional file 2: Appendix S2). In terms of node betweenness, the top two ranked locations were rice fields of Cádiz and Kenitra provinces (nodes 74 and 164 in the interactive map; named “La Janda” and “Gharb” respectively). Several landfills followed these rice fields in betweenness value, i.e. landfills close to Rabat, Seville and Kenitra cities (nodes 139, 66 and 134 respectively). Regarding strength for “out edges”, the top ranked locations were the landfill and irrigated land close to the city of Lleida (also known as Lérida, nodes 19 and 17 respectively). With respect to strength for “in edges”, the top ranked locations were these same nodes from Lleida in reverse order (reflecting mutuality). Non-irrigated land in Lleida city (node 16) ranked third according to both strength measures, being followed by the landfill at Madrid city according to outgoing flights (node 33) and by its closest irrigated land according to incoming flights (node 34). Furthermore, we identified a total of 36 spatial modules, which are shown both in the interactive map (Additional file 1: Appendix S1) and Table S1 (Additional file 2: Appendix S2). Five of these modules involved a single node and 31 contained several nodes of different habitats. Of these, only four did not include any landfill. Each of the remaining 27 spatial modules includes the habitat patches that receive flights from the same landfill (or landfills).

**Fig. 4:**
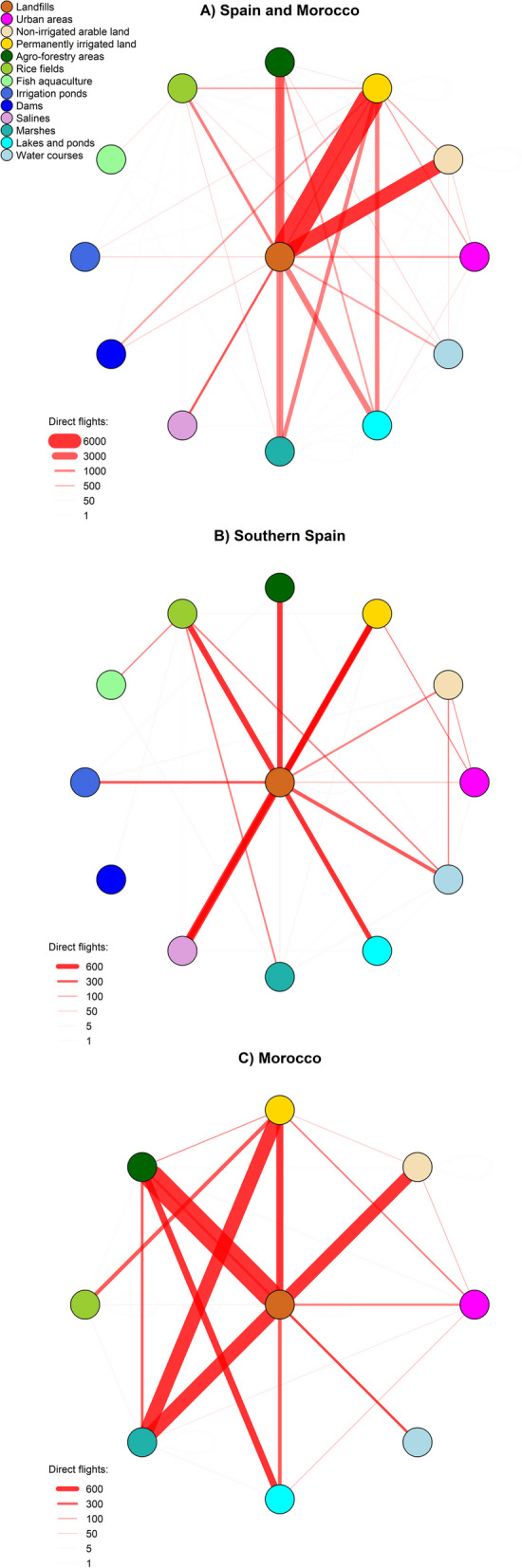
Overall connectivity between different habitat types in the whole study area (**A**), in southern Spain (**B**) and in northern Morocco (**C**). Thickness and colour intensity of links (red lines) are proportional to the number of direct flights between all nodes of the same habitat type

In the undirected network that we built for habitats across the full study area (Fig. 4A), landfills were always involved in the strongest connections. Ranking the links of this network by number of direct flights, the top five ranked connections between habitats were landfills with: permanently irrigated land (10,579 direct flights), non-irrigated arable land (6,112), agro-forestry areas (3,594), marshes (2,721), lakes and ponds (2,442). Importantly, there were clear geographic differences in habitat connectivity provided by non-breeding white storks. The undirected networks constructed for habitats in southern Spain and northern Morocco exhibited marked dissimilarities from one another (Fig. 4B and C), as well as from the entire study area. However, landfills consistently emerged as the most highly connected habitat type.

### Network modelling

The intercept (i.e. “sum”) indicates the expected log-number of direct flights from a default node to another default node, without considering any explanatory variable (Table 2). Because we included node factors for “out” *vs* “in” edges, the estimate given by the intercept applies to the default levels of these factors. For “out edges”, we considered all habitat types except landfills to be default nodes. For “in edges”, we considered default nodes to be landfills, urban areas, non-irrigated arable land, permanently irrigated land and agro-forestry areas. Consequently for the ERGM in southern Spain, the effect estimate of sum indicates that the expected number of direct flights from a default node (all except landfills) to another default node (all except wetlands) is exp(4.75) = 115.

**Table 2:** Summary results of Exponential Random Graph Models (ERGMs) for regional networks in southern Spain and northern Morocco

	Estimate	SE	*z* value	*p* value
*Southern Spain*				
Sum	4.75	0.03	180.82	< 0.001
**Nodeofactor (landfills)**	**1.49**	**0.04**	**33.39**	** < 0.001**
**Nodeifactor (rice fields)**	**2.53**	**0.07**	**38.25**	** < 0.001**
Nodeifactor (fish aquaculture)	− 0.05	0.10	− 0.46	0.645
**Nodeifactor (irrigation ponds)**	**− 2.32**	**0.09**	**− 24.49**	** < 0.001**
**Nodeifactor (dams)**	**− 1.28**	**0.39**	**− 3.29**	** < 0.001**
**Nodeifactor (salines)**	**1.66**	**0.05**	**31.85**	** < 0.001**
**Nodeifactor (marshes)**	**− 1.45**	**0.11**	**− 13.16**	** < 0.001**
Nodeifactor (lakes and ponds)	− 0.06	0.07	− 0.94	0.349
Nodeifactor (water courses)	0.04	0.06	0.66	0.51
Edgecov (distance)	− 0.15	< 0.01	− 67.69	< 0.001
Edgecov (mutuality)	0.85	0.04	23.45	< 0.001
*Northern Morocco*				
Sum	5.56	0.02	315.00	< 0.001
**Nodeofactor (landfills)**	**0.39**	**0.03**	**13.27**	** < 0.001**
Nodeifactor (rice fields)	0.01	0.08	0.14	0.893
**Nodeifactor (marshes)**	**0.08**	**0.03**	**2.59**	**0.01**
**Nodeifactor (lakes and ponds)**	**− 0.28**	**0.04**	**− 6.58**	** < 0.001**
**Nodeifactor (water courses)**	**− 0.78**	**0.06**	**− 11.97**	** < 0.001**
Edgecov (distance)	− 0.19	< 0.01	− 84.38	< 0.001
Edgecov (mutuality)	0.08	0.02	5.03	< 0.001

Both models quantify the effect of landfills as sources of direct flights and wetlands as sinks, taking into account the same covariates (sum, geographical distance between nodes, and mutuality). Significant effects of node factors are highlighted in bold. SE indicates Standard Error. “Nodeofactor” refers to outgoing flights, and “nodeifactor” to incoming flights

We found widespread significant effects for node factors included in both ERGMs. In a manner analogous to any GLM, effect estimates for factor levels here refer to differences with the intercept (i.e. default levels of factors). We found a positive effect of landfills as sources of white stork movements in both ERGMs (Table 2). For our model in southern Spain, the expected number of direct flights from any landfill to any default node is exp(1.49) = 4.5 times higher than the expected number of direct flights given by the intercept. For our model in Morocco, the positive effect of landfills as sources of direct flights was comparatively lower (Table 2). The other effect estimates for node factors can be interpreted in a similar way, in all cases such statements hold in the sense of “all else being equal” (i.e. given no change among values of the other statistics).

In our model for southern Spain, we detected significant positive effects of rice fields and salines as sinks of white stork movements (Table 2). Specifically, the expected number of direct flights from any default node to rice fields is exp(2.53) and to salines is exp(1.66) times higher than the expected number of direct flights given by the intercept. By contrast, we detected significant negative effects of irrigation ponds, dams and marshes as sinks of stork movements in southern Spain; and we did not detect any significant effect of fish aquaculture, lakes and ponds or water courses. In our model for northern Morocco (Table 2), we found a mild but significant positive effect of marshes as a sink for stork movements. On the other hand, we found significant negative effects of lakes and ponds or water courses as sinks of stork movements in Morocco; and the effect of rice fields was not significant.

In both models, we also found significant effects of geographical distance and mutuality. Effect estimates for such link covariates were similar across models. For example considering southern Spain, the negative effect of distance between nodes indicates that for each unit of increase in this variable (i.e. one km), the expected number of direct flights from a default node to another default node decreases exp(−0.15) times. Finally, the estimate for mutuality indicates that the expected number of direct flights from a default node “i” to another default node “j” increases by exp(0.85) times for every negated unit by which link value “i−j” differs from link value “j−i”. Since mutuality is expressed as the negative absolute difference, these units are negated, and so this change will always be a decrease [35].

## Discussion

This study revealed how non-breeding white storks connect different habitat patches scattered from Spain to Morocco. Given their opportunistic behaviour, white storks constitute the perfect study model to understand connections between landfills and other habitats (including terrestrial, aquatic, natural and artificial habitats). We combined a high-quality GPS dataset with a land cover surface to disentangle a spatial network whose nodes were defined by sites within the landscape, and whose links corresponded to non-stop direct flights from one node to another. Our results reveal how landfills were key nodes in the ecological connectivity exerted by white storks. Furthermore, for regional networks in southern Spain and northern Morocco, we characterized the relative importance of landfills acting as sources of flights, and wetlands acting as sinks.

The analysis of landscape connectivity has been used in conservation biology in order to understand habitat fragmentation [18]. In this sense, key habitat patches in the landscape benefit a particular species in some way, such as promoting genetic flux [18], seed dispersal [46] or survival across migratory flyways [34, 43, 61, 67]. By contrast, in our study, the most connected habitat type (i.e. landfills) may have detrimental effects on other habitats used by white storks. Few studies have addressed the importance of birds in landscape connectivity [18, 20, 43, 46] and fewer have addressed the interface between terrestrial and aquatic environments [38]. The increasing collection of GPS datasets [32] facilitates development of this research line. The combination of GPS movements with a land cover surface has often been used to study intensity of habitat use, and nowadays is also applied to connectivity between habitat patches [66]. Network theory provides quantitative metrics to identify key habitat patches, but also statistical models to explain the topology of spatial networks [20, 29, 59]. To the best of our knowledge, ours is the first study applying ERGMs to spatial networks weighted by wildlife movements (but see Belkhiria et al. [9] for nomadic herders). Such models were originally developed for social science and then incorporated into behavioural ecology [33, 59]. In agreement with Fletcher et al. [20], we demonstrate in this study the power of social network models for understanding the factors that determine landscape connectivity.

Distance between nodes has been frequently used as the main or single variable to explain landscape connectivity by network construction. In our study, we explored the effect of node habitat in source-sinks dynamics of movements while controlling for the effect of distance. As expected, results from our ERGMs showed the strong effect of this covariate to explain connectivity among habitat patches [18, 20, 67] (Belkhiria et al. 2019). In this sense, nearby nodes were highly connected and usually included in the same spatial cluster (module), representing daily movements of white storks such as those from foraging to resting sites (thus the strong effect of mutuality in ERGMs). On the other hand, distant nodes were connected by few direct flights, which typically represent migratory movements. Key stopover and wintering sites were identified by either nodes with higher betweenness values (i.e. stepping-stone sites) or by nodes with higher strength values (i.e. main sources and sinks of movements). Consequently, our spatial network and calculated metrics cover different spatial scales and functional movements of our model species. Such integration of movement ecology offers a broad perspective on the role of white storks in connecting habitat patches at different scales, from local foraging trips to long-distance migratory movements. Previous studies have used movement data from GPS-tagged storks and geese to construct a spatial network [34, 61, 67], but they were focused only on long-distance migratory movements. In our study, we aimed to quantify connectivity between sites of different habitat, for which we needed to define nodes at a local geographical scale.

The importance of landfills as a reliable source of food for animals has been widely studied [51], which explain effects on white stork behaviour and population dynamics [4, 7, 19, 24, 62]. Opportunistic species such as white storks and lesser black-backed gulls *Larus fuscus* are able to exploit a wide variety of environments due to their trophic plasticity and adaptability [7, 24, 36]. Both species forage frequently on landfills, which provide constant access to food resources with relatively low energy investment [36, 51, 62]. Wetlands can be then used by both species for resting, drinking or additional foraging, as occurs at rice fields around Doñana National Park [38], i.e. node 68 in our study. However, other important wetland nodes in the spatial network for lesser black-backed gulls (e.g. “Fuente Piedra” lake, Malaga province) are not important for white storks. Thus, each species has its own ecological requirements with different implications, despite sharing a tendency for landfill foraging. In contrast to the spatial network for lesser black-backed gulls [38], in the current study we used a much larger GPS dataset, increased the geographic scope, accounted for more habitat types, and used ERGMs to explain network topology.

Results from both ERGMs showed a positive effect of landfills as sources of direct flights. However, the role of each wetland habitat as a sink for flights differed across regions. Most artificial wetland types (fish aquaculture, irrigation ponds, dams and salines) were not included in the spatial network of Morocco, whereas rice fields and natural wetlands changed their effects substantially across regions. Such differences highlight the opportunistic behaviour of white storks, as they connect habitat patches depending on the spatial configuration of the landscape. In particular, our results suggest that white storks select landfills for foraging, and then frequently visit the closest water body for resting, drinking or additional foraging (as in the case of rice fields). Because our spatial network is directed and our ERGMs tested the effects of landfills as sources, and the effect of wetlands as sinks of flights, our results have implications for the potential of white storks to biovector contaminants from landfills into habitats of conservation concern. Our ERGMs showed significant positive effects of rice fields, salines and marshes as destinations for movements, despite the fact that terrestrial agricultural habitats (considered as default nodes) were involved in the strongest connections between habitat types. Besides white storks and lesser black-backed gulls, there are many other bird species that forage in landfills [3, 47, 51, 60], and their role in ecological connectivity could also be investigated following our methods.

We have been conservative in this study as we filtered only non-stop flights from the GPS dataset. By adopting such criteria, we made our analysis relevant to the biovectoring potential of matter with relatively low retention time within the avian digestive tract, such as seeds (e.g. 2.6 h on average for lesser black-backed gull faeces, ranging from < 1 to 29 h [40]). We can expect similar retention times for other solid objects such as plastic fragments, as their sizes can be comparable to that of some seeds. Considering that most direct flights in our GPS dataset (i.e. 99%) lasted less than two hours, our results are relevant to potential biovectoring by white storks and particularly the dispersal of contaminants. Further studies are required to ground truth such processes in different habitats. Previous studies show that white storks feeding in landfills can transport high amounts of plastics through ingestion and subsequent defecation or regurgitation as pellets [7, 27, 49]. Unpublished data from the authors of this study show that plastics were found in virtually all (158 out of 161) white stork pellets sampled across southern Spain (i.e. nodes 56, 61, 62, 64, 66 and 75, including water courses, landfills, agro-forestry areas, irrigation ponds and salines). Although extensive information exists on plastic ingestion by biota [57], there is little information on how this plastic moves via wildlife across different habitats [58]. White storks provide an excellent model for plastic ingestion [27, 49], and our study quantifies how they move from landfills to other terrestrial and aquatic habitats. Based on our results, we suggest that the salines in Bay of Cádiz (node 75) could be particularly interesting for detailed studies of plastic vectoring, since this habitat showed a strong effect as a destination of flights (ERGM) and it is separated by only 2 km from the closest landfill.

Previous studies show that landfills act as a source of antibiotic resistant genes and bacteria that white storks may disperse elsewhere [28, 31, 44, 50]. However, until now, it remained unclear how strong were the actual connections provided by storks between landfills and other habitats. In particular for southern Spain, landfills were highly connected with saltpans, irrigated lands and rice fields, which may have implications for human health as these are habitats for food production [63]. The potential of antimicrobial resistance dispersal by a given bird (vector) is determined by the retention time of bacteria within its digestive tract, and the distance it may travel during this period [1]. Studies in gulls and mallards have shown carriage times of up to 29 days after experimental inoculation [22, 55]. The spatial network presented in this study illustrates how antibiotic resistant bacteria and genes may disseminate through the landscape. For example, nodes for which we calculated higher betweenness (e.g. rice fields in Cádiz and Kenitra) could be accumulating antibiotic resistance dispersed by storks moving between Spain and Morocco. Interestingly, plastic contamination promotes antimicrobial resistant gene dissemination [8]. The interaction between plastic and antibiotic resistance is an emergent “One-health” research line, and our study quantified the ecological links from landfills (i.e. known pools of both plastics and microbial resistances) to wetlands (i.e. habitats where different bacteria and genes can co-exist and interact [21, 63]).

As well as exploiting landfills, like many other waterbirds white storks have changed their habitat use to exploit wet agricultural environments [52]. During rice harvest (from October to November) abundant food resources become accessible in the rice fields of Seville. At this time, white storks disperse seeds ingested accidentally when foraging on the red swamp crayfish, some of which are from alien plant species [41]. This crayfish is also very common in Gharb rice fields in Morocco [56, 68], where seed dispersal via white storks may occur as well. Moreover, we also identified direct flights from rice fields in southern Spain to nearby fish farms, rivers, marshes, ponds or dams. This finding helps to understand the potential dispersal of aquatic invertebrates [25], since white storks can also disperse crustaceans, bryozoans and snails by gut passage, including the invasive *Physella acuta* [42]. Considering such results together with our spatial network, we suggest that white storks are likely to spread weeds and invasive species between different habitats.

## Conclusions

We deciphered a spatial network for a widespread migratory bird covering more than 1000 km from the Pyrenees to Morocco, and describe it in unparalleled detail. To our knowledge, there is no similar study at such a scale. Our network approach reveals how white storks move from landfills to different terrestrial and aquatic habitats. Such results have further implications for biodiversity conservation and human welfare. By providing connectivity, storks can facilitate global change. Overall, the results of the present study advance our understanding of how bird movements may explain or predict patterns in contaminant concentrations, eutrophication, biological invasions, or the entry of pathogens into food webs. This could help to improve management plans (e.g. landfill design; [53]) to minimise potential biovectoring into natural areas or locations managed for food production. Similar studies are required for other migratory birds using landfills, and further studies on the implications of stork movements (e.g. biovectoring of pollutants and pathogens) could be conducted within the network we identified.

### Supplementary Information

**Additional file 1**. Interactive map showing the spatial network and GPS tracks.

**Additional file 2**. **Table S1.** Summary results for nodes included in the spatially-explicit network of non-breeding white storks (N = 114). The ID of the given location matches Node ID in the interactive map. Latitude and longitude are those of the centroids. Only nodes with at least six individuals in a given non-breeding event are listed here. The convex hull polygons for modules are shown within the interactive map.

## Data Availability

The datasets analysed during the current study are available in Movebank Data Repository, https://www.movebank.org. Specifically, four Movebank studies were used: "LifeTrack White Stork Oberschwaben" (https://www.doi.org/10.5441/001/1.c42j3js7), "LifeTrack White Stork Bavaria" (https://www.doi.org/10.5441/001/1.v1cs4nn0), "LifeTrack White Stork SW Germany” (https://www.doi.org/10.5441/001/1.ck04mn78) and "LifeTrack White Stork Rheinland-Pfalz" (https://www.doi.org/10.5441/001/1.4192t2j4). The R code developed for this study is not novel, and will be stored at DigitalCSIC repository upon acceptance.
