# Supplementary material for: White stork movements reveal the ecological connectivity between landfills and different habitats
Source: Mov Ecol. 2023 Mar 28;11:18. doi: 10.1186/s40462-023-00380-7 (PMC10045253; doi:10.1186/s40462-023-00380-7)
Supplement: Supplementary file 1 — Additional file 1. Interactive map showing the spatial network and GPS tracks. [file 40462_2023_380_MOESM1_ESM.zip › 40462_2023_380_MOESM1_ESM/White stork movements reveal the ecological connectivity - Appendix S1.pdf]

Manuscript title: White stork movements reveal the ecological connectivity between landfills and different habitats.

Author names for publication: Cosme López-Calderón, Víctor Martín-Vélez, Julio Blas, Ursula Höfle, Marta I. Sánchez, Andrea Flack, Wolfgang Fiedler, Martin Wikelski, Andy J. Green.

Supporting Information: Appendix S1.

Interactive map

The enclosed file is an interactive map with summary results and GPS tracks used in this study. This file is an "html widget", that must be opened with a browser. Supported browsers are Google Chrome, Mozilla Firefox and Microsoft Edge (however this file cannot be opened adequately with Internet Explorer neither Opera). To open the html file, it should remain together with "Interactive_map_files" in the same folder. The zoom is set with the mouse wheel and the map can be moved by clicking and panning (alternatively it may be moved by pressing arrows from the keyboard). When placing the mouse over a given element (e.g. line or polygon), it should be highlighted and a specific label should appear. The specific layers are described as follows.

-Nodes: these are the different sites used by the studied white storks. The legend of the map and colours indicate the specific habitat type. A given node may be composed by several polygons (which are highlighted when placing the mouse over). When highlighted, the label shows node ID (from zero to 180) and the number of individuals that used this node in a given non-breeding event (i.e. *bird-years*). All potential nodes used by less than six bird-years were removed from the network and subsequent analyses (see Methods for details).

-Links: these are the connections provided by the studied white storks. We defined a connection between two nodes as a non-stopping flight from the first to the second node (i.e. a direct flight). When highlighted, the label indicates the direction of this link (from the first habitat to the second one) and the number of direct flights in this connection.

-Modules: these are the convex hull polygons including nodes which belong to the same community within the spatial network. We defined the community structure of our network by minimizing the description length of 1000 random walks (see "Methods" for details). When highlighted, the label indicates the ID of the given module (from one to 36).

-Tracks: these are four layers with GPS tracks used in this study. Each of these layers corresponds to a Movebank study from which we downloaded data ("SW Germany", "Oberschwaben", "Bavaria" and "Rheinland-Pfalz"). The filtered samples sizes across Movebank studies were respectively 81, 28, 25, and 65 bird-years. Position fixes in these layers were filtered from raw data as described in "Methods". When highlighted, the label indicates the ID of the specific GPS tag (e.g. "3917") during a given non-breeding event (e.g.

“2015”). We recommend not to select all these layers at the same time, yet the interactive map would render slow.

The user may choose the relative order to show each layer by selecting and deselecting a given layer (i.e. the last layer selected will appear on top). Finally, there are five different base maps to be selected according to personal preferences. “Dark” and “Light” are simplified maps in black *vs* white. The aesthetic of the map is optimized to be used over Dark base map. By contrast, base map “OSM” (Open Street Map) contains many labels which may be helpful to identify places and natural reserves. Base map “Satellite” is the open surface earth layer from ESRI. Base map “Elevation” is an open shaded relief layer from ESRI.
