# Supplementary figures and images for: White stork movements reveal the ecological connectivity between landfills and different habitats
Source: Mov Ecol. 2023 Mar 28;11:18. doi: 10.1186/s40462-023-00380-7 (PMC10045253; doi:10.1186/s40462-023-00380-7)

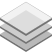

Supplement: Supplementary file 1 — Additional file 1. Interactive map showing the spatial network and GPS tracks. [file 40462_2023_380_MOESM1_ESM.zip › 40462_2023_380_MOESM1_ESM/Interactive_map_files/leaflet-1.3.1/images/layers-2x.png]

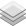

Supplement: Supplementary file 1 — Additional file 1. Interactive map showing the spatial network and GPS tracks. [file 40462_2023_380_MOESM1_ESM.zip › 40462_2023_380_MOESM1_ESM/Interactive_map_files/leaflet-1.3.1/images/layers.png]

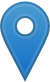

Supplement: Supplementary file 1 — Additional file 1. Interactive map showing the spatial network and GPS tracks. [file 40462_2023_380_MOESM1_ESM.zip › 40462_2023_380_MOESM1_ESM/Interactive_map_files/leaflet-1.3.1/images/marker-icon-2x.png]

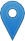

Supplement: Supplementary file 1 — Additional file 1. Interactive map showing the spatial network and GPS tracks. [file 40462_2023_380_MOESM1_ESM.zip › 40462_2023_380_MOESM1_ESM/Interactive_map_files/leaflet-1.3.1/images/marker-icon.png]

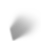

Supplement: Supplementary file 1 — Additional file 1. Interactive map showing the spatial network and GPS tracks. [file 40462_2023_380_MOESM1_ESM.zip › 40462_2023_380_MOESM1_ESM/Interactive_map_files/leaflet-1.3.1/images/marker-shadow.png]

Supplement: Supplementary file 1 — Additional file 1. Interactive map showing the spatial network and GPS tracks. [file 40462_2023_380_MOESM1_ESM.zip › 40462_2023_380_MOESM1_ESM/Interactive_map_files/rstudio_leaflet-1.3.1/images/1px.png]
