# Supplementary material for: White stork movements reveal the ecological connectivity between landfills and different habitats
Source: Mov Ecol. 2023 Mar 28;11:18. doi: 10.1186/s40462-023-00380-7 (PMC10045253; doi:10.1186/s40462-023-00380-7)
Supplement: Supplementary file 2 — Additional file 2. Table S1. Summary results for nodes included in the spatially-explicit network of non-breeding white storks (N = 114). The ID of the given location matches Node ID in the interactive map. Latitude and longitude are those of the centroids. Only nodes with at least six individuals in a given non-breeding event are listed here. The convex hull polygons for modules are shown within the interactive map. [file 40462_2023_380_MOESM2_ESM.pdf]

Manuscript title: White stork movements reveal the ecological connectivity between landfills and different habitats.

Author names for publication: Cosme López-Calderón, Víctor Martín-Vélez, Julio Blas, Ursula Höfle, Marta I. Sánchez, Andrea Flack, Wolfgang Fiedler, Martin Wikelski, Andy J. Green.

Supporting Information: Appendix S2.

Table S1. Summary results for nodes included in the spatially-explicit network of non-breeding white storks (N=114). The ID of the given location matches Node ID in the interactive map. Latitude and longitude are those of the centroids. Only nodes with at least six individuals in a given non-breeding event are listed here. The convex hull polygons for modules are shown within the interactive map.

ID	Lat	Lon	Habitat	Bird-years	Betweenness	Strength out	Strength in	Area (km^2)	Module
1	41.66	0.95	Landfills	57	7	1623	1410	0.16	1
2	41.82	0.77	Landfills	12	2	16	15	0.08	30
3	42.11	2.79	Landfills	11	60	163	169	0.12	7
4	41.62	0.63	Urban areas	59	2	227	297	7.06	1
5	41.79	0.81	Urban areas	15	4	33	26	2.71	30
6	41.68	0.95	Lakes and ponds	31	2	532	398	1.27	1
7	42.11	2.77	Urban areas	16	6	66	56	5.79	7
8	42.31	3.01	Urban areas	8	0	2	7	0.50	10
9	42.30	3.06	Landfills	48	28	457	358	0.40	10
10	42.12	2.75	Non-irrigated land	11	0	169	171	2.77	7
11	42.21	3.06	Rice fields	22	110	83	60	2.10	10
12	42.22	3.10	Marshes	58	158	352	466	10.92	10
13	42.13	2.75	Lakes and ponds	8	0	10	14	1.17	7
14	41.63	0.48	Irrigation ponds	15	0	121	76	0.16	1
16	41.53	0.64	Non-irrigated land	97	3	2356	2183	9.84	1
17	41.58	0.58	Irrigated land	125	253	3422	4895	1425.42	1
18	41.51	0.42	Rice fields	32	0	55	40	1.36	1
19	41.54	0.61	Landfills	101	48	4611	3629	1.36	1
24	41.78	0.04	Rice fields	38	0	7	9	68.07	1
25	42.09	-1.14	Landfills	8	0	229	171	0.25	24
26	41.57	-0.85	Landfills	34	0	63	56	7.00	25
27	41.54	-0.64	Irrigated land	73	36	57	65	497.86	25
28	40.38	-3.62	Urban areas	10	0	41	66	1.09	3
29	40.46	-3.36	Landfills	8	0	1	0	0.71	3
30	39.42	-2.86	Marshes	6	0	0	0	3.66	34
31	40.23	-3.64	Urban areas	26	0	101	95	1.28	29
32	40.26	-3.64	Landfills	41	30	202	163	1.42	29
33	40.33	-3.59	Landfills	51	11	1667	1290	3.52	3
34	40.19	-3.58	Irrigated land	52	0	1354	1594	74.09	3
35	40.30	-3.53	Lakes and ponds	39	9	916	1062	6.60	3
36	40.00	-4.65	Landfills	8	6	160	171	0.06	17
37	39.86	-4.17	Landfills	25	6	89	61	0.29	16
38	39.86	-4.21	Irrigated land	24	0	264	301	11.70	16
39	39.85	-4.28	Dams	26	16	304	298	9.09	16
41	40.03	-4.66	Irrigated land	8	0	132	221	1.02	17
42	40.04	-4.66	Dams	8	0	306	205	4.11	17
46	38.95	-5.71	Landfills	7	0	0	0	0.44	36
47	39.10	-5.73	Rice fields	12	0	0	0	269.69	35
50	39.43	-3.22	Landfills	31	82	151	73	0.64	27
51	39.15	-3.70	Marshes	17	0	0	3	29.68	33

ID	Lat	Lon	Habitat	Bird-years	Betweenness	Strength out	Strength in	Area (km^2)	Module
52	39.46	-3.31	Marshes	31	0	73	148	35.95	27
53	38.94	-3.69	Water courses	13	0	27	27	0.76	20
54	38.86	-3.66	Landfills	33	4	72	39	0.67	20
55	38.77	-3.71	Dams	19	0	12	42	5.77	20
56	37.79	-4.74	Water courses	41	0	249	365	2.53	8
57	37.81	-4.75	Landfills	54	454	340	211	0.65	8
58	37.81	-4.80	Urban areas	6	0	85	86	0.30	8
60	37.30	-5.30	Lakes and ponds	21	282	136	144	2.44	26
61	37.23	-5.37	Landfills	26	391	146	137	0.32	26
62	37.59	-6.00	Agro-forestry areas	26	55	317	367	3.05	9
63	37.58	-6.00	Irrigation ponds	16	0	113	126	0.30	9
64	37.42	-6.65	Irrigation ponds	8	0	52	63	0.19	18
65	37.45	-6.64	Landfills	9	116	183	117	0.39	18
66	37.23	-5.88	Landfills	59	1430	520	230	1.72	2
67	37.60	-6.01	Landfills	28	191	489	425	0.61	9
68	37.13	-6.13	Rice fields	69	1044	462	746	420.88	2
69	37.50	-6.04	Water courses	10	162	7	11	1.12	9
70	36.62	-6.15	Irrigated land	36	69	671	713	38.78	12
71	36.41	-6.11	Landfills	17	0	61	45	0.18	21
72	36.48	-6.01	Landfills	40	874	212	88	0.86	21
73	36.61	-6.12	Landfills	33	151	934	795	1.07	12
74	36.26	-5.88	Rice fields	50	2072	109	82	31.63	14
75	36.57	-6.15	Salines	39	941	785	893	102.61	12
76	36.42	-5.73	Dams	10	0	7	8	23.14	14
77	36.23	-5.48	Landfills	16	58	75	83	0.77	11
78	36.17	-5.48	Irrigated land	15	58	147	129	4.96	11
79	36.17	-5.44	Marshes	8	0	12	24	1.06	11
80	36.44	-6.08	Lakes and ponds	36	39	122	240	1.37	21
83	41.62	0.60	Lakes and ponds	45	4	135	160	0.06	1
84	42.09	3.12	Rice fields	9	28	105	100	9.79	23
85	42.06	-1.18	Rice fields	8	0	171	229	46.80	24
86	37.15	-5.90	Marshes	14	0	9	10	0.26	2
88	37.42	-6.60	Non-irrigated land	8	0	83	137	10.92	18
89	35.74	-5.76	Landfills	6	0	101	95	0.44	15
90	35.75	-5.77	Non-irrigated land	6	182	92	95	2.32	15
91	36.20	-5.90	Fish aquaculture	19	1085	62	97	8.71	14
92	42.06	3.07	Irrigated land	8	0	97	104	8.09	23
93	35.75	-5.80	Urban areas	7	56	46	48	6.76	15
95	35.60	-5.96	Marshes	10	513	3	2	52.21	32
99	35.16	-6.16	Landfills	7	65	30	35	0.17	13
100	35.16	-6.16	Non-irrigated land	6	0	32	27	0.06	13
101	35.00	-5.97	Landfills	18	513	818	737	0.04	4
102	35.16	-6.11	Marshes	7	643	9	12	20.81	13
103	35.11	-6.05	Rice fields	11	150	4	5	25.45	13
104	34.97	-6.01	Irrigated land	14	0	231	318	43.41	4
106	35.00	-5.90	Urban areas	9	0	12	33	0.44	4

ID	Lat	Lon	Habitat	Bird-years	Betweenness	Strength out	Strength in	Area (km^2)	Module
107	35.00	-5.98	Agro-forestry areas	20	122	823	779	3.08	4
111	40.23	-3.66	Non-irrigated land	14	1	70	80	10.92	29
112	34.67	-5.99	Landfills	9	67	450	461	0.24	19
114	34.68	-6.00	Urban areas	7	0	88	66	2.15	19
115	34.59	-5.90	Landfills	8	48	299	292	0.19	28
116	34.60	-5.90	Non-irrigated land	6	3	287	275	0.50	28
133	34.31	-6.54	Irrigated land	22	1143	1109	1175	27.42	6
134	34.28	-6.57	Landfills	22	1350	1235	1146	0.27	6
136	34.28	-6.56	Urban areas	19	144	190	200	2.55	6
137	34.01	-6.81	Marshes	6	1	2	2	1.28	31
138	34.00	-6.80	Non-irrigated land	7	119	4	3	4.94	31
139	33.87	-6.81	Landfills	13	1474	1266	1120	0.83	5
140	33.90	-6.86	Agro-forestry areas	13	0	1374	1566	36.27	5
161	37.81	-4.79	Non-irrigated land	17	0	154	164	1.08	8
162	37.20	-5.88	Irrigation ponds	6	0	2	7	0.03	2
163	34.62	-6.14	Irrigated land	29	388	319	361	604.11	22
164	34.57	-6.28	Rice fields	32	1639	252	240	145.02	22
165	34.66	-5.99	Non-irrigated land	8	0	439	440	0.05	19
167	36.22	-5.44	Urban areas	11	0	94	90	0.16	11
169	37.21	-6.19	Lakes and ponds	23	0	29	24	2.10	2
171	37.05	-6.30	Marshes	35	261	115	127	403.41	2
172	37.14	-6.22	Water courses	22	4	113	114	17.24	2
173	36.95	-6.23	Fish aquaculture	11	0	13	13	27.89	2
176	35.02	-5.99	Marshes	16	0	393	404	1.98	4
177	34.26	-6.54	Lakes and ponds	20	0	135	167	4.14	6
178	34.28	-6.58	Marshes	19	0	1511	1492	0.78	6
179	33.89	-6.79	Lakes and ponds	8	1	580	553	0.64	5
180	33.87	-6.82	Water courses	8	0	301	284	0.05	5
